# Author Correction: Magnetoreception in a freshwater ciliate arises from endosymbiosis

**DOI:** 10.1038/s41467-026-74787-2

**Published:** 2026-06-23

**Authors:** Romain Bolzoni, Caroline L. Monteil, Béatrice Alonso, Marine Bergot, Daniel M. Chevrier, Christian Godon, Nicolas Menguy, Stephanie Fouteau, Violette Da Cunha, Fériel Skouri-Panet, Eva Pereiro, Arnaud Duverger, David Vallenet, Corinne Cruaud, Fernanda Abreu, Karim Benzerara, Christopher T. Lefevre

**Affiliations:** 1 Aix-Marseille Université, CEA, CNRS, UMR7265, BIAM Biosciences and Biotechnologies Institute of Aix-Marseille, CEA Cadarache, Saint-Paul-lez-Durance, France; 2 UMR CNRS 7590, IMPMC Institut de Minéralogie, de Physique des Matériaux et de Cosmochimie, MNHN, IRD, Sorbonne Université, Paris, France; 3 LABGeM, Génomique Métabolique, Genoscope, Institut François Jacob, CEA, CNRS, Université d’Évry, Université Paris-Saclay, Evry, France; 4 ALBA Synchrotron Light Source, Cerdanyola del Vallés, Barcelona, Spain; 5 Genoscope, Institut de biologie François Jacob, CEA, Université Paris-Saclay, Evry, France; 6 Instituto de microbiologia Paulo de Goés, Universidade Fedral do Rio de Janeiro, Rio de Janeiro, Brazil

**Keywords:** Phylogenetics, Water microbiology, Microbiome, Symbiosis

Correction to: *Nature Communications* 10.1038/s41467-026-70462-8, published online 10 March 2026

In the version of the article initially published, in the Discussion, both instances of “*Protisticella dordonia*” have been corrected to “*Protisticella dordognensis*” and one instance of “*Endodesulfobacter magneticum*” has been corrected to “*Endodesulfobacter magneticus*”. These species names have now been corrected in the HTML and PDF versions of the article and the Supplementary information.

