# Supplementary material for: Magnetoreception in a freshwater ciliate arises from endosymbiosis
Source: Nat Commun. 2026 Mar 10;17:3732. doi: 10.1038/s41467-026-70462-8 (PMC13103400; doi:10.1038/s41467-026-70462-8)
Supplement: Supplementary file 1 — Supplementary Information [file 41467_2026_70462_MOESM1_ESM.pdf]

Supplementary information

Magnetoreception in a freshwater ciliate arises from endosymbiosis

Romain Bolzoni[1,2†], Caroline L. Monteil[1†*], Béatrice Alonso[1], Marine Bergot[1], Daniel M. Chevrier[1], Christian Godon[1], Nicolas Menguy[2], Stephanie Fouteau[3], Violette da Cunha[3], Fériel Skouri-Panet[2], Eva Pereiro[4], Arnaud Duverger[2], David Vallenet[3], Corinne Cruaud[5], Fernanda Abreu[6], Karim Benzerara[2], Christopher T. Lefevre[1*]

[1]Aix-Marseille Université, CEA, CNRS, BIAM, UMR7265, Institut de Biosciences et Biotechnologies Aix-Marseille, CEA Cadarache, F-13115, Saint-Paul-lez-Durance, France

[2]Sorbonne Université, UMR CNRS 7590, MNHN, IRD, Institut de Minéralogie, de Physique des Matériaux et de Cosmochimie, IMPMC, 75005, Paris, France

[3]LABGeM, Génomique Métabolique, CEA, Genoscope, Institut François Jacob, CNRS, Université d'Évry, Université Paris-Saclay, Evry, France

[4]ALBA Synchrotron Light Source, Cerdanyola del Vallés, Barcelona 08290, Spain

[5]Genoscope, Institut de biologie François Jacob, CEA, Université Paris-Saclay, Evry, France

[6]Instituto de microbiologia Paulo de Goés, Universidade Fedral do Rio de Janeiro, Rio de Janeiro, Brazil

[†] These authors contributed equally to this work

*Corresponding authors: Caroline L. Monteil and Christopher T. Lefevre

Emails: caroline.monteil@cea.fr; christopher.lefevre@cea.fr

1. Supplementary Figures

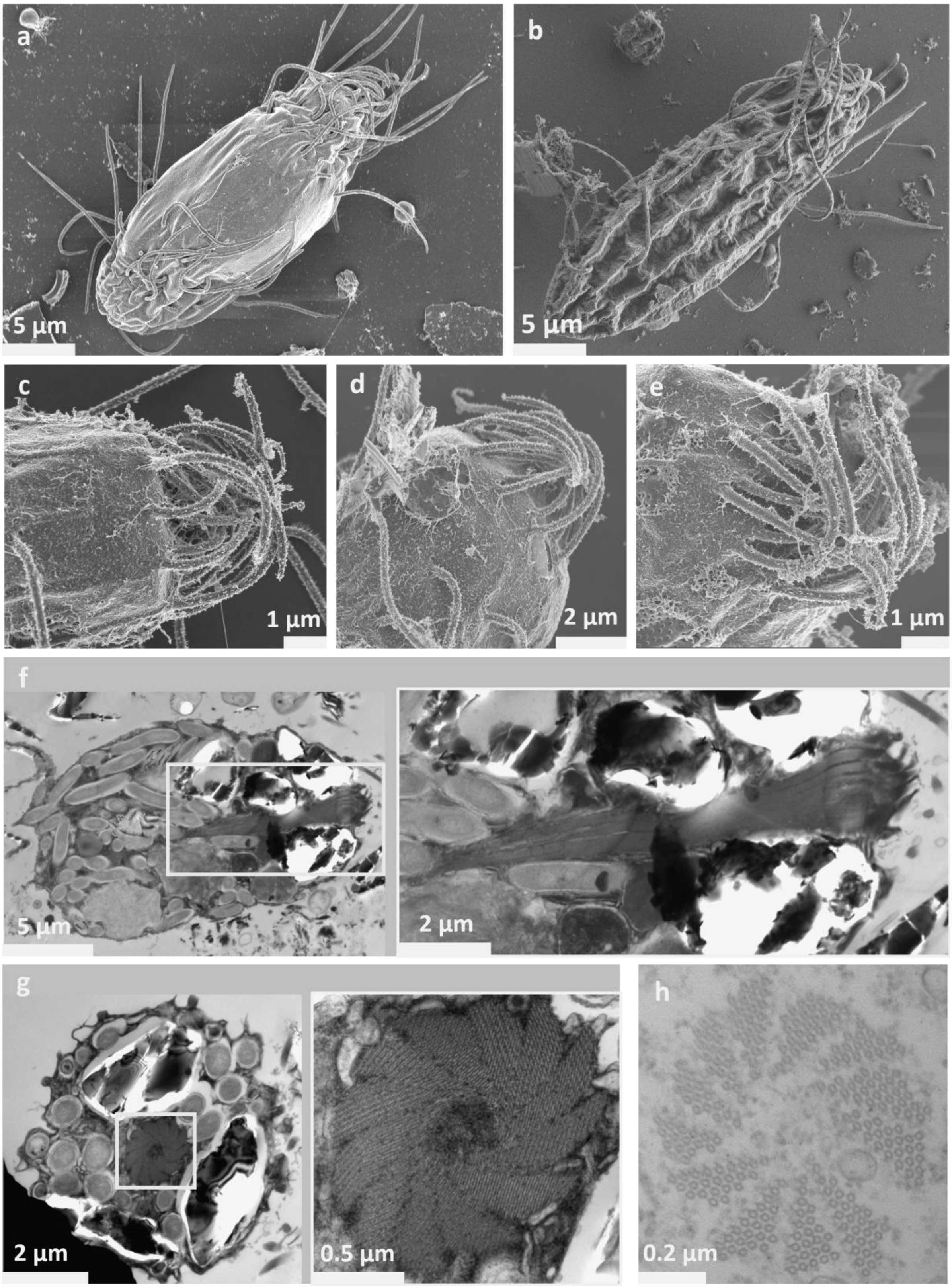

Supplementary Figure 1. Electron microscopy images of magnetotactic protists showing the structural organization of the ciliate body. a and **b,** Scanning electron microscopy (SEM) images of magnetotactic protists showing the cilia organized in three zones. **c** to **e**, SEM images of the frontal part of magnetotactic protists showing the oral bulge surrounded by cilia with a membranelle-like organization. **f,** Transmission electron microscope (TEM) images of a longitudinal thin-section of a

magnetotactic protist showing the structure of the cytostome that occupy half of the protist body, the white frame in the panel on the left corresponds to the panel on the right. **g** and **h**, TEM images of vertical thin-section of the frontal part of magnetotactic protists showing the organization in twelve sets of microtubules of the cytostome (in **g,** the frame in the panel on the left corresponds to the panel on the right).

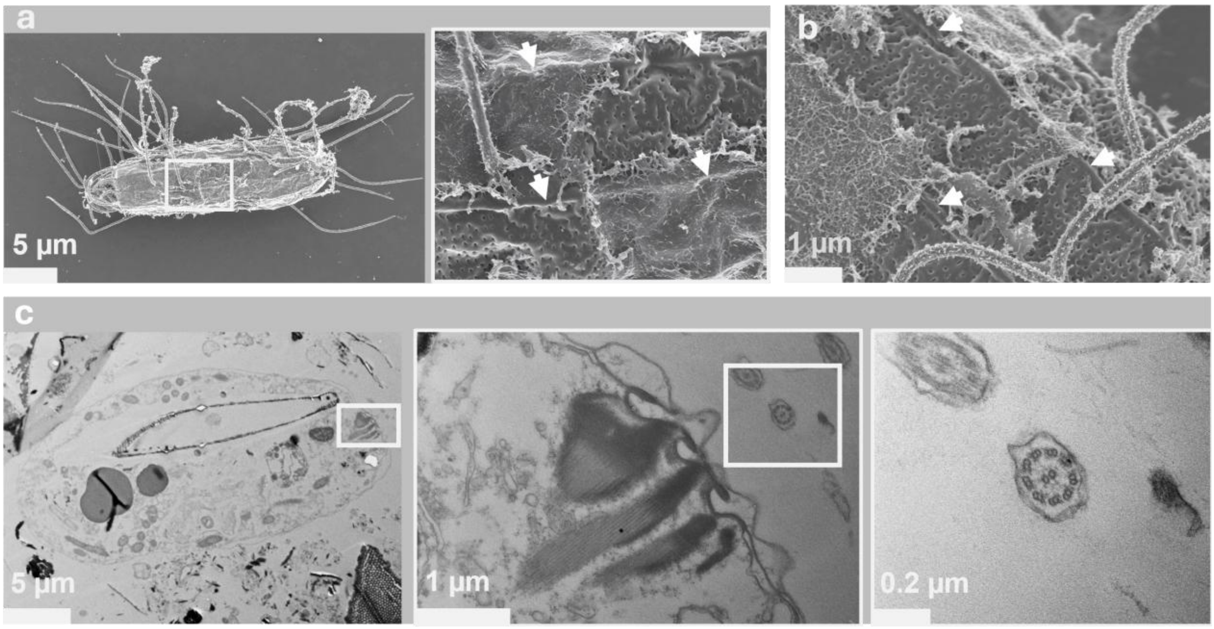

Supplementary Figure 2. Electron microscopy images of the magnetotactic protists showing the structure of the ciliate envelope and cilia. a and **b,** Scanning electron microscopy images showing a mucus-like surrounding the entire protist and, below this envelop, pores visible in the inset of **a** and **b** panels. Tubular structures (shown with white arrows) are also observed beneath the cell membrane likely maintaining the shape of the cell. **c,** Transmission electron microscopy images of a thin-sectioned magnetotactic protist showing a canonical '9 + 2' microtubule axoneme structure of the cilia. The frame in the image of the panel on the left corresponds to the image in the panel on the middle, the frame in the image of the panel in the middle corresponds to the image in the panel on the right.

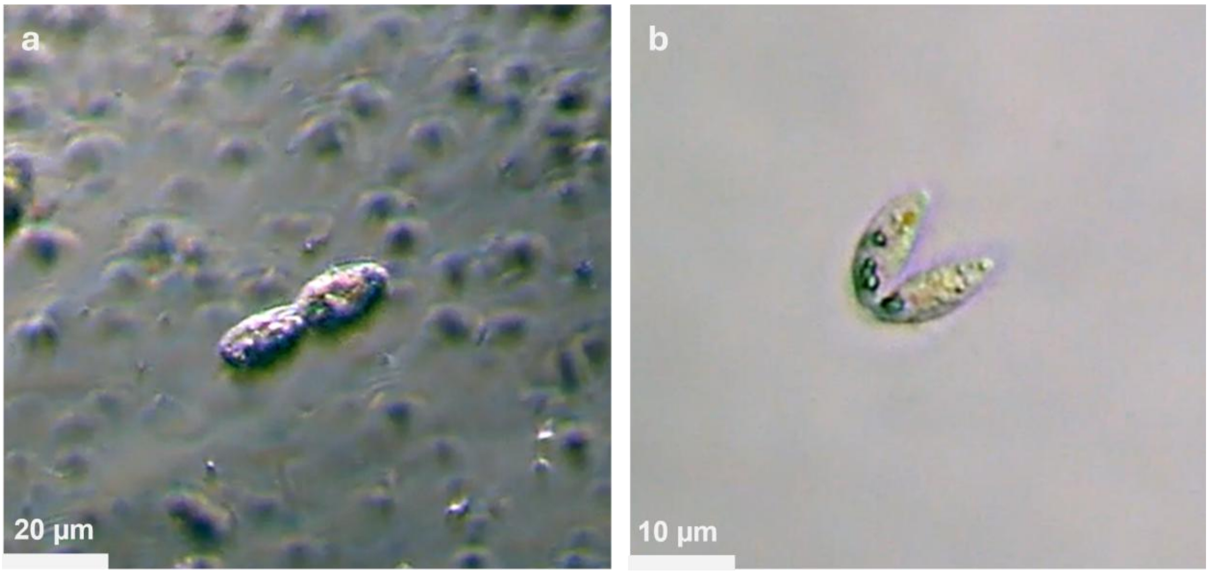

Supplementary Figure 3. Light microscopy images of magnetotactic protists showing their potentials multiplication strategies. a, Two dividing cells. **b,** Reproduction of two protists.

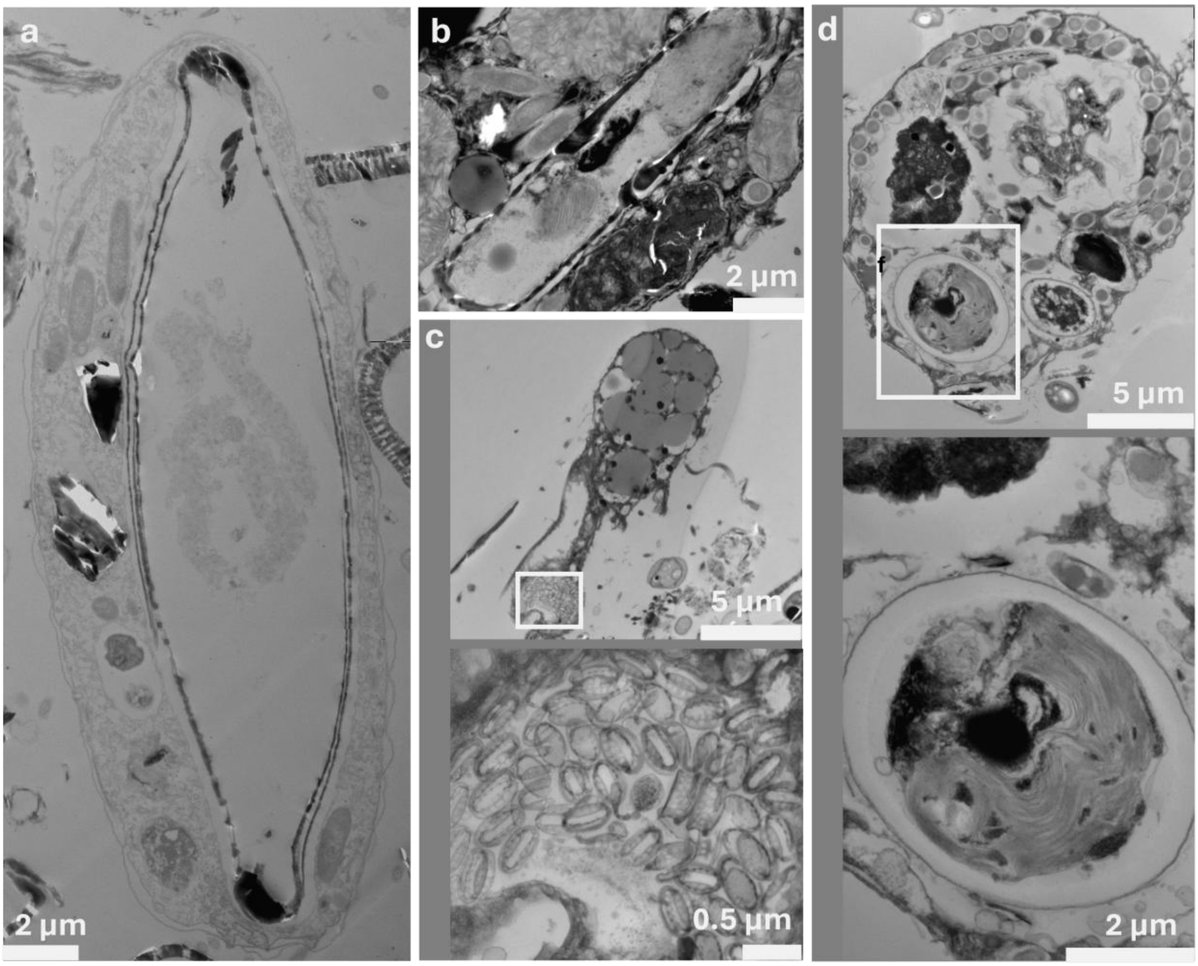

Supplementary Figure 4. Evidence of the presence of various photosynthetic microorganisms inside the magnetotactic protists. A and b, Transmission electron microscopy (TEM) images of thin-sectioned magnetotactic protists showing the presence of a diatom occupying a large part of the cytoplasm. **c,** TEM images of a thin-sectioned magnetotactic protist partially exploded containing a vacuole with numerous structures resembling calcium carbonate scales formed by coccolithophores affiliated to the *Pleurochrysis* genus. **d**, TEM images of a magnetotactic protist containing a vacuole with a structure resembling to *Chlamydomonas* or *Chlorella* microalgae.

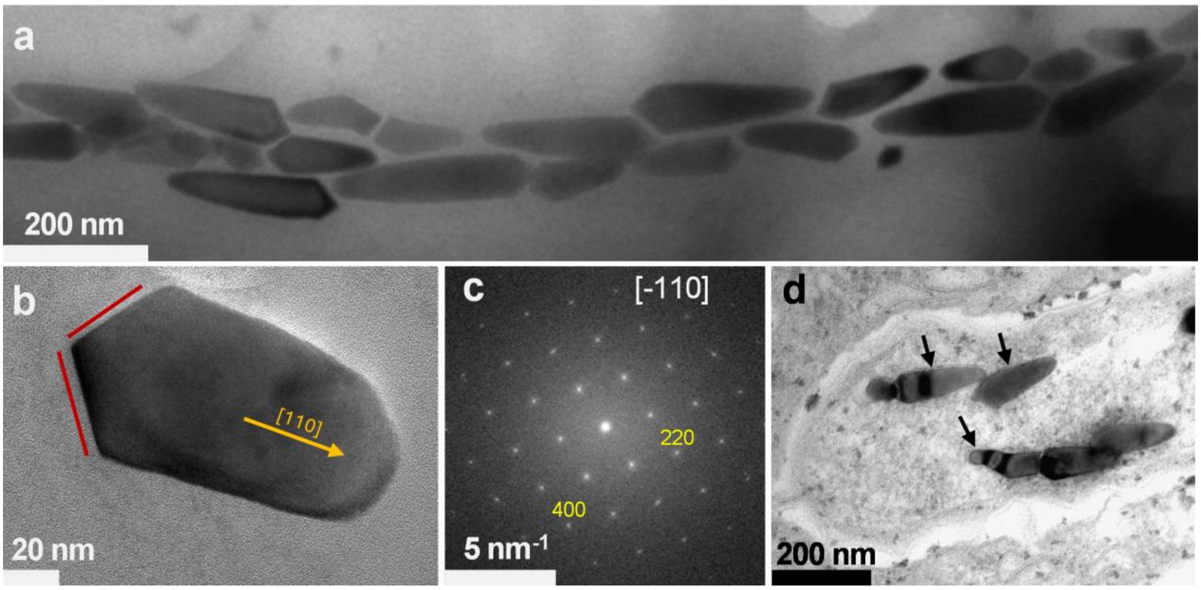

Supplementary Figure 5. Characterization of magnetosome-like particles present in some intracellular vesicles of the magnetotactic protist. **a,** TEM image of a bundle of bullet-shaped particles present in an endosymbiotic bacterium showing some particles longer than 200 nm. **b,** High-resolution TEM image of a single magnetosome biomineralized by an endosymbiotic bacterium (red lines represent the {111} plane; the yellow arrow represents the [110] direction of elongation) and **c,** corresponding diffraction indexed with the magnetite structure ([-110] shows the direction of observation). **d,** TEM image of a longitudinal thin section of an endosymbiont showing a membrane (black arrows) surrounding the magnetite particles.

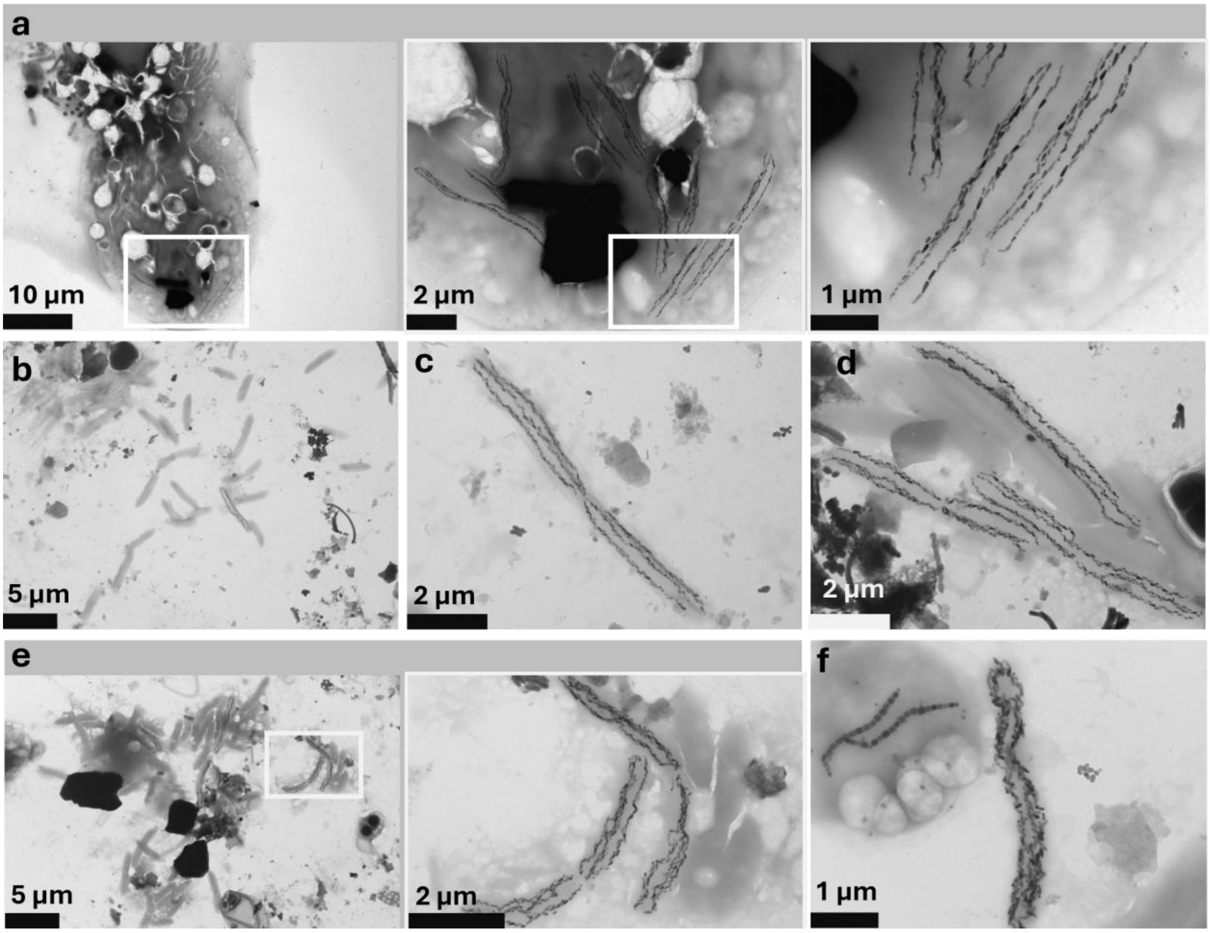

Supplementary Figure 6. Transmission electron microscopy (TEM) images of magnetotactic protists samples in different freshwater environments. a, TEM images of a magnetotactic protist isolated from Lake Aydat, Auvergne. The white frame on the left panel corresponds to the image on the panel in the middle. The white frame on the panel in the middle corresponds to the image on the panel on the right. **b** to **d,** TEM images of an exploded magnetotactic protist showing several endosymbiotic bacteria with or without magnetosomes, isolated from a river in Ploemeur, Britany. **e** and **f,** TEM images of an exploded magnetotactic protist showing several endosymbiotic bacteria with or without magnetosomes, isolated from Lake Lannénec, Britany.

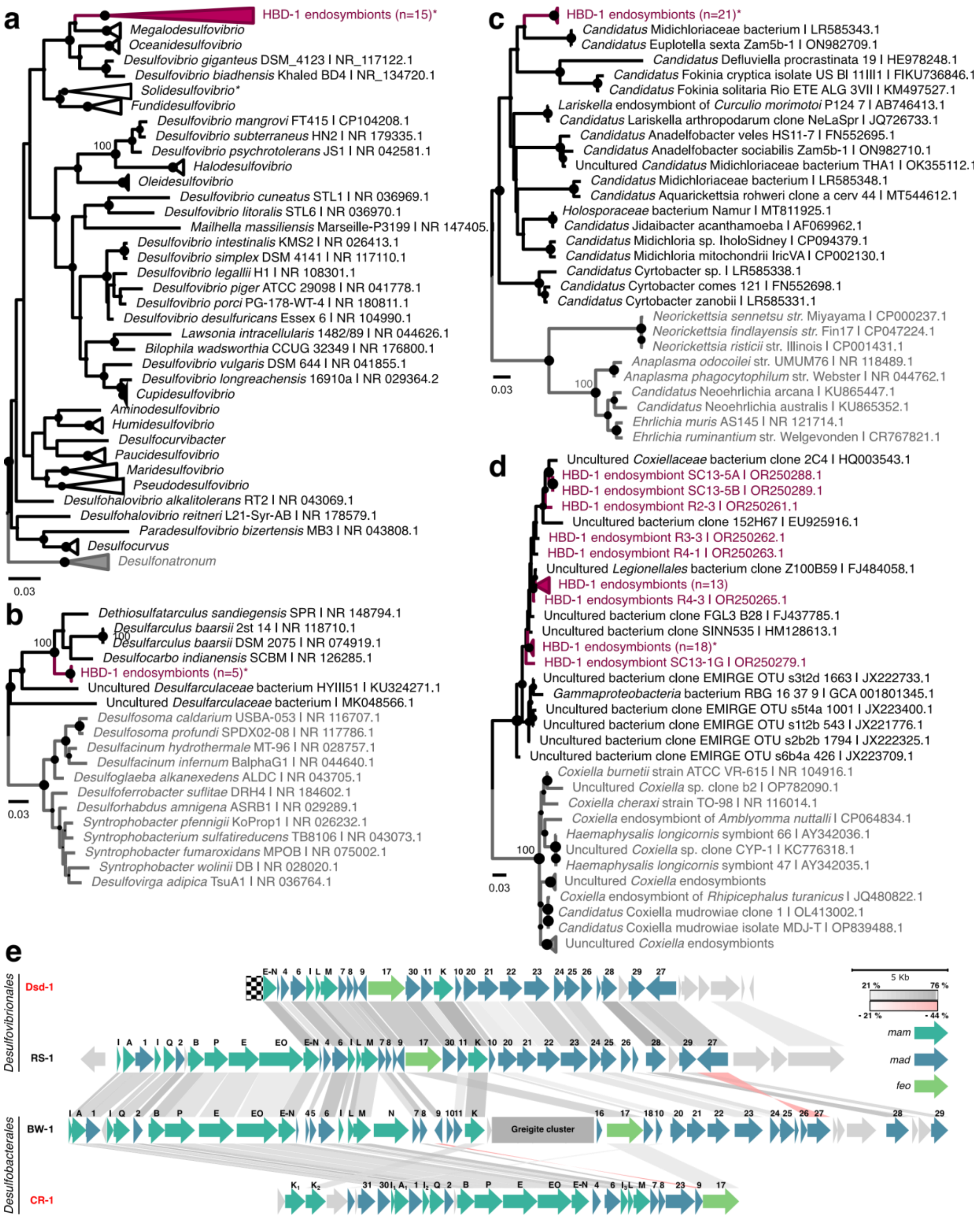

Supplementary Figure 7. Phylogenetic trees based on the 16S rRNA gene sequence showing the relationships of symbionts with other environmental bacteria and analysis of the magnetosome gene cluster (MGC) assembled within the hologenome. a to **d,** Maximum-Likelihood trees of the *Desulfovibrionaceae* family (taxonomy NCBI), *Desulfarculaceae* family (taxonomy NCBI), *Ca.* Midichloriaceae family (taxonomy NCBI) and *Coxiellaceae* family (taxonomy NCBI), showing relationships of DsD-1, GD-1, DcD-1 and MD-1, respectively. Sequences were retrieved from genomes, and from the NCBI database (https://www.ncbi.nlm.nih.gov, July 2023). Trees were constructed using the maximum likelihood method implemented in IQ-TREE[1]. The support was computed by the UFBootstrap method (1000 replicates) implemented in IQ-TREE . Values are represented by circles on internal nodes and their size is proportional to the bootstrap value. The symbionts sequences have been deposited in Genbank (OR294250-OR294271, OR250250-OR250289, OR294289-OR294298, and

OR294333-OR294370) and the corresponding accession numbers are given in the sequence names. **e**, Comparative analysis of magnetosome gene clusters from reference genomes of *Desulfobacterota* (*i.e.*, strains RS-1[3], BW-1[4] and the magnetic ectosymbiont CR-1[5]) with the genome of the endosymbiotic biomineralizing bacterium DsD-1. Each arrow represents a *mam* or *mad* gene conserved in magnetotactic *Desulfobacterota*[4]. Grey arrows are genes of unknown function or not conserved in MTB. Gene syntenies are organized according to the taxonomic affiliation. The chequer box in DsD-1 synteny marks the contig truncation which suggests a complete RS-1-like MGC in DsD-1. No homologs to greigite specific *mam* genes were detected in DsD-1. Note that syntenies were drawn to scale, except for the greigite region of strain BW-1 that was collapsed. Sequence identities between Reciprocal Best Hits (RBH) were estimated with MMseqs2[6] and are represented by bands, with their intensity reflecting the percentage of identity. Some homologs are not linked to due to high sequence divergence and/or the presence of multiple paralogs. Homologues families were then determined by the presence of conserved domains using the Microscope platform[7]. Comparison of magnetosomes genes in assembled from the Dsd-1 genome with those of the reference strains *Solidesulfovibrio magneticus* RS-1 and *Fundidesulfovibrio magnetotacticus* FSS-1 are given in Supplementary Data 5.

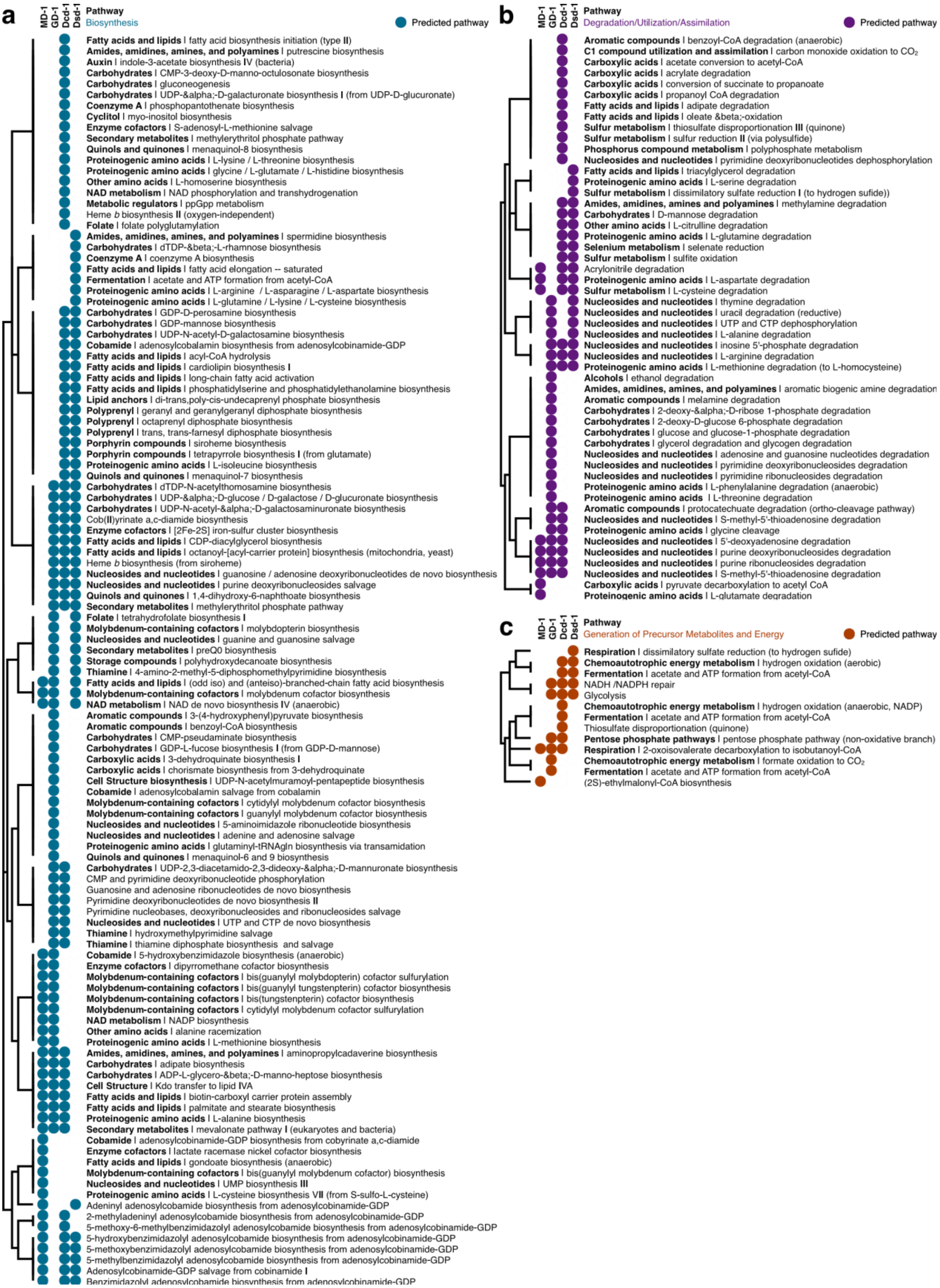

Supplementary Figure 8. Heatmaps representing the occurrence of selected predicted metabolic pathways and functions in the four symbionts of the magnetotactic ciliate HBD-1. **a** to **c**, Comparative analysis of MetaCyc[8] metabolic pathways predicted with the Pathway Tools software in at least one of the symbiont genomes classified in Biosynthesis (**a**), Degradation/Utilization/Assimilation (**b**) and Generation of Precursor Metabolites and Energy (**c**). Each pathway is partially associated to its

ontology in MetaCyc database. The full analysis and pathway ontology is given in Supplementary Data 10. Metabolic pathways are organized based on a hierarchical clustering analysis (Euclidean distance clustering algorithm) according to their pair-wise distance. Absence of prediction can be linked to the absence of a single reaction / enzyme / gene mandatory for the pathway prediction. Yet this absence can be a false negative and be linked to the quality of the draft genome assembly.

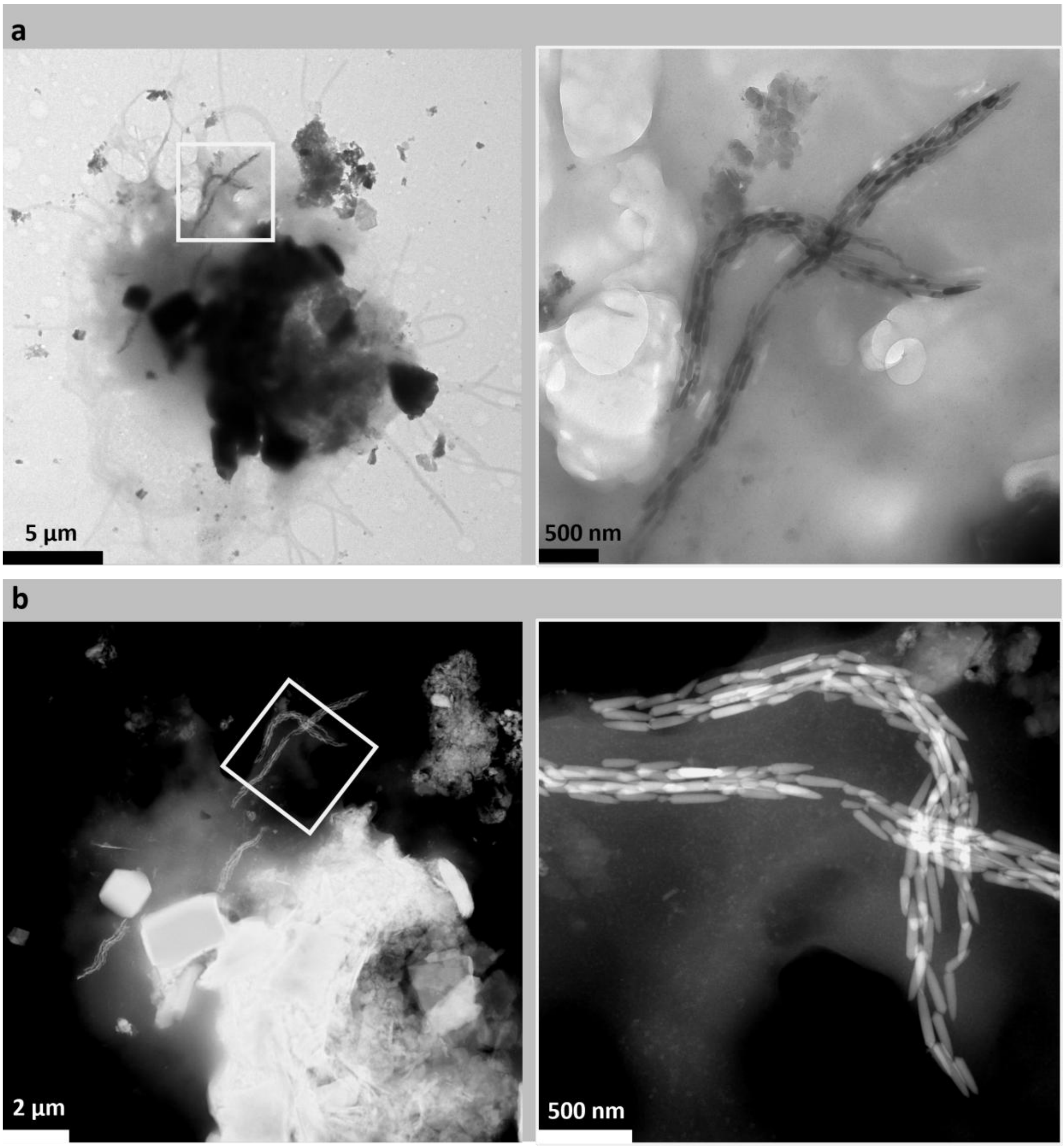

Supplementary Figure 9. Electron microscopy images of a single-celled eukaryote magnetically enriched from Ururaí River, RJ, Brazil. Images were obtained on a Tecnai G20 FEG microscope (FEI, Hillsboro, OR) operated at 200 kV in scanning TEM (STEM) and HRTEM modes with a Fischione high-angle annular dark field (HAADF) detector model M3000, and a 4 k × 4 k Gatan UltraScan 1000 CCD camera. **a,** TEM images, **b,** STEM-HAADF images of the same magnetotactic protist showing the presence of magnetosomes-containing endosymbiotic bacteria similar to those observed in France. White frames on the left panels correspond to the images on the right panels.

2. Supplementary Tables

Supplementary Table 1
Oligonucleotide FISH probes used to authenticate the 16S rRNA gene sequences of the different endosymbiotic bacteria

Probes	Specificity	Sequence (5'-3') of probe	Position
**EUB633p**	Most Bacteria	ATTO633-GCTGCCTCCCGTAGGAGT	322-339
**HBD1-ALPHAp**	*Alphaproteobacteria* endosymbionts	Alexa555-CTAAGGCCTTCATCATTCACGCGACGTC	375-402
**HBD1-GAMMA1p**	*Gammaproteobacteria* endosymbionts	ATTO488-CCTGGGATTTCAAGAGTAGG	969-988
**HBD1-GAMMA1-2p**	*Gammaproteobacteria* endosymbionts	ATTO633-CCTGGGATTTCAAGAGTAGG	969-988
**HBD1-DARCU1-2p**	*Desulfarculia* endosymbionts	ATTO425-CATCTGACTTACAGGTCCGCCTACACG	562-588
**HBD1-DELTAp**	*Desulfovibrionia* endosymbionts	ATTO488-CCGTCAGCCTAAAGGCCTTTC	465-485

3. Supplementary Results

Supplementary Results 1. Taxon description according to the rules of the SeqCode. Names were deposited under the SeqCode Registry Accession number r:dgcae3s5. Because the genome quality of DcD-1 did not meet SeqCode criteria, the *Candidatus* status was assigned to *Desulfella intracellularis* instead.

Description of *Protisticellales* ord. nov.

*Protisticella*les (Pro.tis.ti.cel.la'les. N.L. fem. n. *Protisticella*, type genus of the order; L. fem. pl. n. suff. *-ales*, ending to denote an order; N.L. fem. pl. n. *Protisticellales*, the order of the genus *Protisticella*)

The type genus of the order is *Protisticella*.

Description of *Protisticellaceae* fam. nov.

Protisticellaceae (Pro.tis.ti.cel.la'ce.ae. N.L. fem. n. *Protisticella*, type genus of the family; L. fem. pl. n. suff. *-aceae*, ending to denote a family; N.L. fem. pl. n. *Protisticellaceae*, the family of the genus *Protisticella*)

The type genus of the family is *Protisticella*.

Description of *Protisticella* gen. nov.

Protisticella (Pro.tis.ti.cel'la. N.L. neut. pl. n. *Protista*, protists, referring to the host; L. fem. n. *cella*, a store-room and in biology a cell; L. fem. n. *Protisticella*, a small bacterium associated with protists)

The description is the same as for the type species *Protisticella dordognensis*.

Description of *Protisticella dordognensis* sp. nov.

Protisticella dordognensis (dor.do.gnen'sis L. fem. n. *Dordognensis*, named after the Dordogne River (France), where the host and endosymbionts were collected)

The type genome is GCA_964213355.1.

Endosymbiotic rod-shaped bacteria of a freshwater magnetotactic ciliate from the Dordogne River (France). Hybridize with the specific oligonucleotide probe HBD1-GAMMA1p (Supplementary Table 1). Assigned to the *Protisticellaceae* family *(Gammaproteobacteria* class; *Protisticellales* order) based on phylogenomic analysis (Fig. 5d).

Description of *Midichloriella* gen. nov.

Midichloriella (Mi.di.chlo.ri.el'la. N.L. fem. n. *Midichloria*, derived from the midichlorians, organisms within the fictional Star Wars universe ; L. fem. dim. n. suff. -*ella,* diminutive ending)

The description is the same as for the type species *Midichloriella endociliophora*.

Description of *Midichloriella endociliophora* sp. nov.

Midichloriella endociliophora (en.do.ci.li.o.pho'ra. Gr. pref. *endo*-, inside; L. fem. *ciliophora*, name of a protist phylum; N.L. fem. adj. *endociliophora*, inside ciliophora, referring to the endosymbiotic nature of this organism within an organism belonging to the *Ciliophora*)

The type genome is GCA_964213345.1.

Endosymbiotic rod-shaped bacteria of a freshwater magnetotactic ciliate. Hybridize with the specific oligonucleotide probe HBD1-ALPHAp (Supplementary Table 1). Assigned to the *Midichloriaceae* family *(Alphaproteobacteria* class ; *Rickettsiales* order) based on phylogenomic analysis (Fig. 5c).

Description of *Endodesulfobacter* gen. nov.

Endodesulfobacter (En.do.de.sul.fo.bac'ter. G. fem. n. *endo*, within; L. prep. *de*, from; L. neut. n. *sulfur*, sulfur (S); N.L. pref. *desulfo*-, desulfuricating (prefix used to characterize a dissimilatory sulfate-reducing procaryote) (in compound words); N.L. masc. n. *bacter*, rod or staff; N.L. masc. n. *Endodesulfobacter*, an endosymbiotic rod-shaped sulfate-reducing bacterium)

The description is the same as for the type species *Endodesulfobacter magneticus*.

Description of *Endodesulfobacter magneticus* sp. nov

Endodesulfobacter magneticus (mag.ne'ti.cus. L. masc. adj. magneticus, magnetic)

The type genome is GCA_965112965.1.

Endosymbiotic rod-shaped bacterium of a freshwater magnetotactic ciliate forming magnetite magnetosomes. Hybridize with the specific oligonucleotide probe HBD1-DELTAp (Supplementary Table 1). Assigned to the *Desulfovibrionaceae* family *(Desulfobacterota* phylum ; *Desulfovibrionia* class) based on phylogenomic analysis (Fig. 5a).

4. References

1. Minh, B. Q. *et al.* IQ-TREE 2: new models and efficient methods for phylogenetic inference in the genomic era. *Molecular Biology and Evolution* **37**, 1530–1534 (2020).

2. Hoang, D. T., Chernomor, O., von Haeseler, A., Minh, B. Q. & Vinh, L. S. UFBoot2: Improving the Ultrafast Bootstrap Approximation. *Mol Biol Evol* **35**, 518–522 (2018).

3. Nakazawa, H. *et al.* Whole genome sequence of *Desulfovibrio magneticus* strain RS-1 revealed common gene clusters in magnetotactic bacteria. *Genome Res.* **19**, 1801–1808 (2009).

4. Lefèvre, C. T. *et al.* Comparative genomic analysis of magnetotactic bacteria from the *Deltaproteobacteria* provides new insights into magnetite and greigite magnetosome genes required for magnetotaxis. *Environmental Microbiology* **15**, 2712–2735 (2013).

5. Monteil, C. L. *et al.* Ectosymbiotic bacteria at the origin of magnetoreception in a marine protist. *Nat Microbiol* **4**, 1088–1095 (2019).

6. Steinegger, M. & Söding, J. MMseqs2 enables sensitive protein sequence searching for the analysis of massive data sets. *Nat Biotechnol* **35**, 1026–1028 (2017).

7. Vallenet, D. *et al.* MicroScope: an integrated platform for the annotation and exploration of microbial gene functions through genomic, pangenomic and metabolic comparative analysis. *Nucleic Acids Research* **48**, D579–D589 (2020).

8. Caspi, R. *et al.* The MetaCyc database of metabolic pathways and enzymes - a 2019 update. *Nucleic Acids Res* **48**, D445–D453 (2020).

9. Néron, B. *et al.* MacSyFinder v2: improved modelling and search engine to identify molecular systems in genomes. *Peer Community Journal* **3**, (2023).

10. Zhou, S., Liu, B., Zheng, D., Chen, L. & Yang, J. VFDB 2025: an integrated resource for exploring anti-virulence compounds. *Nucleic Acids Res* gkae968 (2024) doi:10.1093/nar/gkae968.

11. Joensen, K. G. *et al.* Real-time whole-genome sequencing for routine typing, surveillance, and outbreak detection of verotoxigenic Escherichia coli. *J Clin Microbiol* **52**, 1501–1510 (2014).
