# Supplementary material for: Magnetoreception in a freshwater ciliate arises from endosymbiosis
Source: Nat Commun. 2026 Mar 10;17:3732. doi: 10.1038/s41467-026-70462-8 (PMC13103400; doi:10.1038/s41467-026-70462-8)
Supplement: Supplementary file 2 — Description of Additional Supplementary Files [file 41467_2026_70462_MOESM2_ESM.pdf]

Description of Additional Supplementary Data

Magnetoreception in a freshwater ciliate arises from endosymbiosis

Romain Bolzoni[1,2†], Caroline L. Monteil[1†*], Béatrice Alonso[1], Marine Bergot[1], Daniel M. Chevrier[1], Christian Godon[1], Nicolas Menguy[2], Stephanie Fouteau[3], Violette da Cunha[3], Fériel Skouri-Panet[2], Eva Pereiro[4], Arnaud Duverger[2], David Vallenet[3], Corinne Cruaud[5], Fernanda Abreu[6], Karim Benzerara[2], Christopher T. Lefevre[1*]

[1]Aix-Marseille Université, CEA, CNRS, BIAM, UMR7265, Institut de Biosciences et Biotechnologies Aix-Marseille, CEA Cadarache, F-13115, Saint-Paul-lez-Durance, France

[2]Sorbonne Université, UMR CNRS 7590, MNHN, IRD, Institut de Minéralogie, de Physique des Matériaux et de Cosmochimie, IMPMC, 75005, Paris, France

[3]LABGeM, Génomique Métabolique, CEA, Genoscope, Institut François Jacob, CNRS, Université d'Évry, Université Paris-Saclay, Evry, France

[4]ALBA Synchrotron Light Source, Cerdanyola del Vallés, Barcelona 08290, Spain

[5]Genoscope, Institut de biologie François Jacob, CEA, Université Paris-Saclay, Evry, France

[6]Instituto de microbiologia Paulo de Goés, Universidade Fedral do Rio de Janeiro, Rio de Janeiro, Brazil

[†] These authors contributed equally to this work

*Corresponding authors: Caroline L. Monteil and Christopher T. Lefevre

Emails: caroline.monteil@cea.fr; christopher.lefevre@cea.fr

1. Supplementary Data

Supplementary Data 1
Phylogenetic position of the magnetotactic protist. Full and uncollapsed maximum-likelihood tree based on the 18S rRNA gene sequences showing the evolutionary relationships of the magnetotactic ciliates with other *Ciliophora* classes with the full PR^2 accession numbers and taxonomy. See Fig. 1d for the exact same legend. Different colors distinguish different *Ciliophora* classes. The scale represents the substitution rate. A SH-aLRT branch test and ultrafast bootstrap approximation estimated from 1000 replicates were conducted to provide branch support values. A newick version is available in the Supplementary Data 2.

See the Supplementary Data 1 file (.PDF).

Supplementary Data 2
Maximum-likelihood tree based on the 18S rRNA gene sequences showing the evolutionary relationships of the magnetotactic ciliates with other *Ciliophora* classes.
See the Supplementary Data 2 file (.txt).

Supplementary Data 3
Maximum-likelihood tree based on the 16S rRNA gene sequences showing the evolutionary relationships of the four endosymbionts within the *Desulfovibrionaceae*, family, *Desulfarculaceae* family, *Ca*. Midichloriaceae family and UBA9339 order.
See the Supplementary Data 3 file (.txt).

Supplementary Data 4
List of genomes sequenced in this study, the replicon information and the completeness estimated with CheckM2.
See the Supplementary Data 4 file (XLSX).

Supplementary Data 5
Partial magnetosome gene cluster assembled from the Dsd-1 genome and the corresponding syntenic genes in the reference strains *Solidesulfovibrio magneticus* RS-1 and *Fundidesulfovibrio magnetotacticus* FSS-1. Homologs were identified using the MicroScope platform. The MinLrap value represents the length (in amino acids) of the region aligned by BLASTP between the queried DsD-1 sequence and the corresponding subject sequences in RS-1 or FSS-1. It is estimated by dividing the match length by the minimum of the query of subject length. Together with percentage identity and gene synteny, it helps assess whether matched proteins are true orthologs.
See the Supplementary Data 5 file (XLSX).

Supplementary Data 6
Functional classification of protein-coding genes classified by eggNOG-mapper into Cluster of Orthologous Groups (COG) for symbiotic species compared to few free-living MTB and non-MTB species of the same phylum. The colour shade represents the frequency of protein-coding genes for symbionts DsD-1, GD-1, MD-1 and DcD-1 and few free-living species, including *Pseudomonas syringae* B728a | GCA_000012245.1 (Ps), the magnetotactic gammaproteobacterium sp. BW-2 | GCA_008375315.1 (BW-2), *Desulfarculus baarsii* DSM 2075 | GCA_000143965.1 (Db), *Solidesulfovibrio magneticus* RS-1 | GCA_000010665.1 (Sm) and *Rhodospirillum rubrum* ATCC 11170 | GCA_000013085.1 (Rr). A relative color scheme uses the minimum and maximum values in each column to convert values to colors.
See the Supplementary Data 6 file (XLSX).

Supplementary Data 7

Protein-coding genes of the symbionts classified into the Cluster of Orthologous Group "Replication, recombination and repair" by the EGGNOG classification. eggNOG version 5.0.2 is a public database of orthology relationships, gene evolutionary histories and functional annotations. An Orthologous Group (OG) is defined as a cluster of three or more homologous sequences that diverge from the same speciation event. eggNOG-mapper version 2.1.12 was used to transfer functional information from precomputed Orthologous Groups (OGs) from the EggNOG database to novel sequences of the symbionts.
See the Supplementary Data 7 file (XLSX).

Supplementary Data 8
List of proteins containing leucine-rich repeats (LRRs) and ankyrin repeats. Homology searches were performed using the InterPro database (http://www.ebi.ac.uk/interpro/) integrating together predictive models or 'signatures' representing protein domains, families and functional sites from multiple, diverse source databases: Gene3D, PANTHER, Pfam, PIRSF, PRINTS, ProDom, PROSITE, SMART, SUPERFAMILY and TIGRFAMs.
See the Supplementary Data 8 file (XLSX).

Supplementary Data 9
Macromolecular systems found in the GD-1 genome. Macromolecular systems (cellular machinery, metabolic pathways) were searched in GD-1 protein dataset with the program MacSyFinder v2.1.2[9]. In prokaryotes, these systems have often evolutionarily conserved properties: they are made of conserved components, and are encoded in compact loci (conserved genetic architecture). Its detection methods work by searching components of the systems by sequence similarity using Hidden Markov Models (HMM) profiles and analyzing the content and organization of the system.
See the Supplementary Data 9 file (XLSX).

Supplementary Data 10
Protein-coding genes of the symbionts classified into the Cluster of Orthologous Group "Cell motility" by the EGGNOG classification. eggNOG version 5.0.2 is a public database of orthology relationships, gene evolutionary histories and functional annotations. An Orthologous Group (OG) is defined as a cluster of three or more homologous sequences that diverge from the same speciation event. eggNOG-mapper version 2.1.12 was used to transfer functional information from precomputed Orthologous Groups (OGs) from the EggNOG database to novel sequences of the symbionts.
See the Supplementary Data 10 file (XLSX).

Supplementary Data 11
List of putative virulence factors identified in endosymbionts genomes. Reciprocal Best Hits (Identity > 30%) are obtained by running BLASTP on organism proteins against MicroScope virulence database built upon VFDB[10] and VirulenceFinder[11] data. VFDB virulence factor classification has been completed as best as possible with new terms and gene associations. The table is organized into 2 categories: VFDB experimentally demonstrated data and VirulenceFinder.
See the Supplementary Data 11 file (XLSX).

Supplementary Data 12
Comparative analysis of metabolic pathways predicted in at least one of the endosymbiotic genomes based on the MetaCyc pathways database. MetaCyc pathways are supported by the Pathway tools software developed by Peter Karp and his team at SRI international. These pathways were predicted using the PathoLogic module which computes an initial set of pathways by comparing a genome annotation to the metabolic reference database MetaCyc. Black and white boxes represent "predicted" and "not predicted" pathways. They do not reflect a pathway completion rate. Absence of prediction can be linked to the absence of a single reaction/enzyme/gene judged mandatory for the pathway realization.

Yet this absence can be a false negative and be linked to the quality of the draft genome assembly. The KoFamScan output listing genes in symbionts genomes with an assigned KEGG Orthology (KO) that passed adaptive score thresholds and that was used for pathways reconstruction are given in Supplementary Data 13.
See the Supplementary Data 12 file (XLSX).

Supplementary Data 13
KoFamScan output listing genes in endosymbiont genomes with an assigned KEGG Orthology (KO). Each gene name from the fasta files annotated with PROKKA is linked to a KO number, a functional annotation and a HMM E-value / Score. Only matches that passed adaptive score thresholds set for that KO family are shown. The KO identifiers can be mapped to KEGG pathway maps, BRITE hierarchies, and modules. Only matches that passed adaptive score thresholds were used for pathways reconstruction.
See the Supplementary Data 13 file (XLSX).

2. Supplementary Videos

Supplementary Video 1
Light microscope movie of north-seeking magnetotactic microorganisms sampled from the Dordogne River, France. The cells were observed at the edge of a hanging drop close to which the south pole of a magnet was applied. The edge of the drop is covered with numerous magnetotactic bacteria along with magnetotactic protists. At the twelth second, the polarity of the magnet is reversed, inducing the reversion of the swimming direction of all magnetotactic cells.
See the Supplementary Video 1 file (.AVI).

Supplementary Video 2
Light microscope movie of cells aggregated at the edge of a hanging drop, moving collectively under the influence of a rotating magnetic field. The cells were observed at the edge of a hanging drop close to which the south pole of a magnet was applied. Magnetotactic protists are constantly swimming back and forth aligned along the magnetic field lines. Contrasted granules in the anterior pole of cells under focus can be seen.
See the Supplementary Video 2 file (.AVI).

Supplementary Video 3
Light microscope movie of north-seeking magnetotactic microorganisms between slide and cover slide. The cell was observed between slide and coverslip which causes a quick modification of its shape, *i.e.*, the cell rounded and started to break apart few minutes after its deposition on the slide.
See the Supplementary Video 3 file (.MP4).

Supplementary Video 4
Light microscope movie of north-seeking magnetotactic microorganisms sampled from a spring in Ploemeur, Britany, France. The cells were observed at the edge of a hanging drop close to which the south pole of a magnet was applied. The magnetotactic protists performed long run and reverse at the edge of the drop. Magnetotactic cocci are also present at the edge of the drop.
See the Supplementary Video 4 file (.MP4).

Supplementary Video 5
***Z-stacks movie of images acquired with the transmitted light of the CLSM**. The video shows the presence of contrasted granules in the anterior pole (bottom right) of the cell and thin contrasted lines in the first third of the anterior pole of the cell.

See the Supplementary Video 5 file (.AVI).

Supplementary Video 6
Light microscopy movie of a cell observed between slide and coverslip for several minutes. At the eighth seconde, the movie is cut to show the cell exploding few minutes later. Diatoms along with other inclusions and thin rod-shaped structures are seen once the cells disintegrates.
See the Supplementary Video 6 file (.MP4).

Supplementary Video 7
3D reconstructed volume of a magnetotactic protist from cryo-soft X-ray tomography (cryo-SXT). Video depicts the volume contents by traversing the Z-direction. Four magnetosome-producing bacteria (shown with the numbers in their vinicity that appear and disappear with the cells) and an intracellular diatom could be observed in this volume reconstruction.
See the Supplementary Video 7 file (.AVI).

Supplementary Video 8
CLSM 3D-reconstruction of the different z-stack images of a magnetotactic protist. CLSM 3D-reconstruction of the different z-stack (16 slices every 6 µm) images of a magnetotactic protist after fluorescent *in situ* hybridization (FISH) protocol using four different specific oligonucleotide probes of the *Desulfarculia* (blue), the *Desulfovibriona* (green), the *Gammaproteobacteria* (red) and the *Alphaproteobacteria* (yellow).
See the Supplementary Video 8 file (.MP4).

Supplementary Video 9
CLSM 3D-reconstruction of the different z-stack images of three different magnetotactic protists. CLSM 3D-reconstruction of the different z-stack images of three magnetotactic protists after fluorescent *in situ* hybridization (FISH) protocol using four different specific oligonucleotide probes of the *Desulfarculia* (blue), the *Desulfovibriona* (green), the *Gammaproteobacteria* (red) and the *Alphaproteobacteria* (yellow). Note in the third movie the autofluorescence of inclusions at the same wavelength than the fluorochrome (Alxa555, represented in yellow) used to locate the *Alpahproteobacteria*.
See the Supplementary Video 9 file (.MP4).

Supplementary Video 10
Light microscopy movie of the edge of a hanging drop prepared from a magnetically enriched sample from Ubatiba River, Rio de Janeiro, Brazil. The sample was observed by differential interference contrast on a Zeiss Axioimager microscope. Note the presence of both bacteria and a single-celled eukaryote at the edge of the hanging drop. Focus variation allows the observation of black elongated structures (arrowhead) in the eukaryotic cell, which are assumed to be the magnetosomes chain.
See the Supplementary Video 10 file (.MP4).
