# Supplementary figures and images for: Magnetoreception in a freshwater ciliate arises from endosymbiosis
Source: Nat Commun. 2026 Mar 10;17:3732. doi: 10.1038/s41467-026-70462-8 (PMC13103400; doi:10.1038/s41467-026-70462-8)

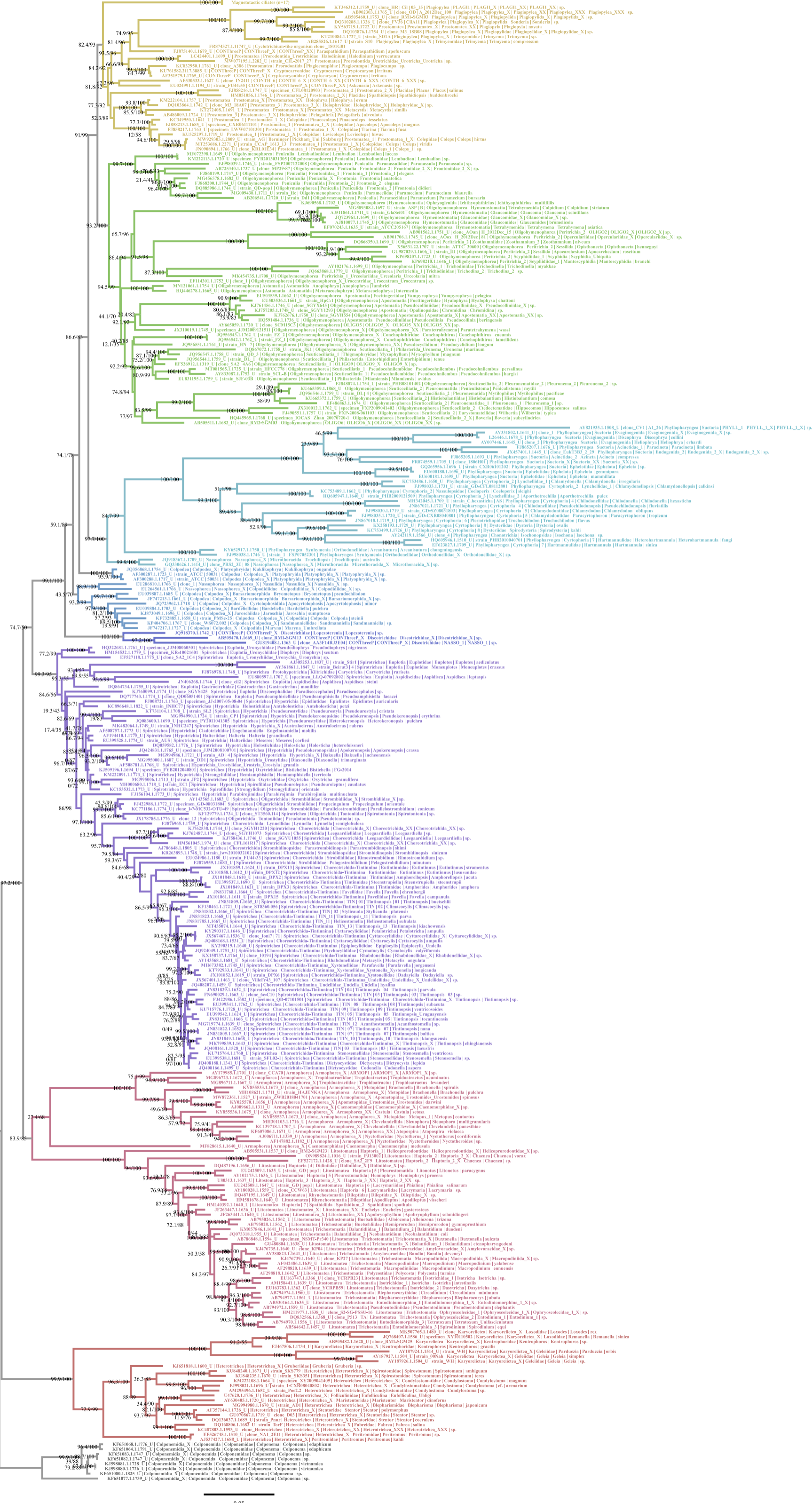

Supplement: Supplementary file 3 — Supplementary Data 1 [file 41467_2026_70462_MOESM3_ESM.pdf]
