# Supplementary material for: Magnetoreception in a freshwater ciliate arises from endosymbiosis
Source: Nat Commun. 2026 Mar 10;17:3732. doi: 10.1038/s41467-026-70462-8 (PMC13103400; doi:10.1038/s41467-026-70462-8)
Supplement: Supplementary file 26 — Reporting Summary [file 41467_2026_70462_MOESM26_ESM.pdf]

nature portfolio

Corresponding author(s): Caroline Monteil and Christopher lefevre

Last updated by author(s): Christopher lefevre

Reporting Summary

Nature Portfolio wishes to improve the reproducibility of the work that we publish. This form provides structure for consistency and transparency in reporting. For further information on Nature Portfolio policies, see our Editorial Policies and the Editorial Policy Checklist.

Statistics

For all statistical analyses, confirm that the following items are present in the figure legend, table legend, main text, or Methods section.

n/a	Confirmed	
☐	☒	The exact sample size (*n*) for each experimental group/condition, given as a discrete number and unit of measurement
☐	☒	A statement on whether measurements were taken from distinct samples or whether the same sample was measured repeatedly
☐	☒	The statistical test(s) used AND whether they are one- or two-sided<br>*Only common tests should be described solely by name; describe more complex techniques in the Methods section.*
☐	☒	A description of all covariates tested
☒	☐	A description of any assumptions or corrections, such as tests of normality and adjustment for multiple comparisons
☐	☒	A full description of the statistical parameters including central tendency (e.g. means) or other basic estimates (e.g. regression coefficient) AND variation (e.g. standard deviation) or associated estimates of uncertainty (e.g. confidence intervals)
☐	☒	For null hypothesis testing, the test statistic (e.g. *F*, *t*, *r*) with confidence intervals, effect sizes, degrees of freedom and *P* value noted<br>*Give P values as exact values whenever suitable.*
☒	☐	For Bayesian analysis, information on the choice of priors and Markov chain Monte Carlo settings
☒	☐	For hierarchical and complex designs, identification of the appropriate level for tests and full reporting of outcomes
☒	☐	Estimates of effect sizes (e.g. Cohen's *d*, Pearson's *r*), indicating how they were calculated

Our web collection on statistics for biologists contains articles on many of the points above.

Software and code

Policy information about availability of computer code

Data collection	GenBank<br>MetaCyc<br>HMMs<br>Pathway/Genome<br>eggnog v 5.0.2<br>Virulence Factor Database (VFDB)<br>PR2 database
Data analysis	BLAST<br>MAFFT v7.490<br>IQ-TREE v2.2<br>ModelFinder<br>FigTree v1.4.4<br>Gblocks v 0.91<br>PythonCyc API v2.0.2<br>SPAdes v.3.13.0<br>CheckM2 v.1.0.2<br>POBE_MATCH<br>Etomo

Zen 3.6
ImageJ2, version 2.16.0/1.54
MinKNOW v.4.5.4 and Guppy v.5.1.13 + b292f4d13
Anvi'o
Bowtie 2
MicroScope platform
RNAmmer
GTDB-Tk v2.1.1
PROKKA v1.14.6
PyHMMER 0.8.0
BMGE v1.12
KofamScan v.1.3.0
Pathway Tools
eggNOG-mapper version 2.1.12
VirulenceFinder

For manuscripts utilizing custom algorithms or software that are central to the research but not yet described in published literature, software must be made available to editors and reviewers. We strongly encourage code deposition in a community repository (e.g. GitHub). See the Nature Portfolio guidelines for submitting code & software for further information.

Data

Policy information about availability of data

All manuscripts must include a data availability statement. This statement should provide the following information, where applicable:

- Accession codes, unique identifiers, or web links for publicly available datasets
- A description of any restrictions on data availability
- For clinical datasets or third party data, please ensure that the statement adheres to our policy

Data generated or analysed during this study are included in this published article and its supplementary information files. The sequencing data were deposited on public databases. The 16S rRNA gene amplicon sequences were deposited in the NCBI Genbank database under the accession numbers OR342250-OR342267, OR294250-OR294271, OR250250-OR250289, OR294289-OR294298, and OR294333-OR294370. The genome assemblies were deposited in the NCBI BioProject database under the accession number PRJEB65892.

Research involving human participants, their data, or biological material

Policy information about studies with human participants or human data. See also policy information about sex, gender (identity/presentation), and sexual orientation and race, ethnicity and racism.

Reporting on sex and gender	Not relevant for this manuscript
Reporting on race, ethnicity, or other socially relevant groupings	Not relevant for this manuscript
Population characteristics	Not relevant for this manuscript
Recruitment	Not relevant for this manuscript
Ethics oversight	Not relevant for this manuscript

Note that full information on the approval of the study protocol must also be provided in the manuscript.

Field-specific reporting

Please select the one below that is the best fit for your research. If you are not sure, read the appropriate sections before making your selection.

☐ Life sciences ☐ Behavioural & social sciences ☒ Ecological, evolutionary & environmental sciences

For a reference copy of the document with all sections, see nature.com/documents/nr-reporting-summary-flat.pdf

Life sciences study design

All studies must disclose on these points even when the disclosure is negative.

Sample size	Samples were collected in 1 L bottles filled with one third of sandy sediments and completed to full capacity with water from the site to exclude air bubbles.
Data exclusions	Not relevant for this manuscript

Replication	Not relevant for this manuscript
Randomization	Not relevant for this manuscript
Blinding	Not relevant for this manuscript

Behavioural & social sciences study design

All studies must disclose on these points even when the disclosure is negative.

Study description	Not relevant for this manuscript
Research sample	Not relevant for this manuscript
Sampling strategy	Not relevant for this manuscript
Data collection	Not relevant for this manuscript
Timing	Not relevant for this manuscript
Data exclusions	Not relevant for this manuscript
Non-participation	Not relevant for this manuscript
Randomization	Not relevant for this manuscript

Ecological, evolutionary & environmental sciences study design

All studies must disclose on these points even when the disclosure is negative.

Study description	We report a new type of symbiosis occurring in freshwater anoxic sediments between ciliate protists and endosymbiotic bacteria.
Research sample	Sediment samples were collected mainly from the shore of the river Dordogne at Beaulieu-sur-Dordogne, Nouvelle-Aquitaine, France (44.9783°N, 1.8378°E) at different seasons between August 2021 and January 2023.
Sampling strategy	Samples were collected from the shore of the river.
Data collection	Most data were obtained thanks to physico-chemical analyses, microscopies observation, gene and genome sequencing. Data were aquired by all the authors.
Timing and spatial scale	Data collection were performed between August 2021 and January 2023 in various aquatic environments in France.
Data exclusions	Not relevant for this manuscript
Reproducibility	All attempts to repeat the experiment were successful. All experiments were independently performed at least three times with similar results. Microscopy observations (including optical, confocal, electron, and X-ray–based imaging) were also conducted on at least three independent occasions, yielding consistent qualitative observations across replicates.
Randomization	Not relevant for this manuscript
Blinding	Not relevant for this manuscript

Did the study involve field work? ☒ Yes ☐ No

Field work, collection and transport

Field conditions	All data were obtained from samples collected from freshwater environments.
Location	Unnamed spring (47°45'13.7"N, 3°28'54.1"W)<br>lake Lannénec (47°44,29.9", 3°28'58.7"W) in Britany<br>Lake Aydat in Auvergne (45°39'54.1"N, 2°58'53.1"E)<br>Cère River (44°55'1.3"N, 1°50'18.8"E)
Access & import/export	All samples were carried out in public areas where no permit or local authorization were required.

Disturbance	Not relevant for this manuscript

Reporting for specific materials, systems and methods

We require information from authors about some types of materials, experimental systems and methods used in many studies. Here, indicate whether each material, system or method listed is relevant to your study. If you are not sure if a list item applies to your research, read the appropriate section before selecting a response.

Materials & experimental systems

n/a	Involved in the study
☒	☐ Antibodies
☒	☐ Eukaryotic cell lines
☒	☐ Palaeontology and archaeology
☒	☐ Animals and other organisms
☒	☐ Clinical data
☒	☐ Dual use research of concern
☒	☐ Plants

Methods

n/a	Involved in the study
☒	☐ ChIP-seq
☒	☐ Flow cytometry
☒	☐ MRI-based neuroimaging

Antibodies

Antibodies used	Not relevant for this manuscript
Validation	Not relevant for this manuscript

Eukaryotic cell lines

Policy information about cell lines and Sex and Gender in Research

Cell line source(s)	Not relevant for this manuscript
Authentication	Not relevant for this manuscript
Mycoplasma contamination	Not relevant for this manuscript
Commonly misidentified lines (See ICLAC register)	Not relevant for this manuscript

Palaeontology and Archaeology

Specimen provenance	Not relevant for this manuscript
Specimen deposition	Not relevant for this manuscript
Dating methods	Not relevant for this manuscript

☐ Tick this box to confirm that the raw and calibrated dates are available in the paper or in Supplementary Information.

Ethics oversight	Not relevant for this manuscript

Note that full information on the approval of the study protocol must also be provided in the manuscript.

Animals and other research organisms

Policy information about studies involving animals; ARRIVE guidelines recommended for reporting animal research, and Sex and Gender in Research

Laboratory animals	Not relevant for this manuscript
Wild animals	Not relevant for this manuscript
Reporting on sex	Not relevant for this manuscript
Field-collected samples	Not relevant for this manuscript

Ethics oversight	Not relevant for this manuscript

Note that full information on the approval of the study protocol must also be provided in the manuscript.

Clinical data

Policy information about clinical studies
All manuscripts should comply with the ICMJE guidelines for publication of clinical research and a completed CONSORT checklist must be included with all submissions.

Clinical trial registration	Not relevant for this manuscript
Study protocol	Not relevant for this manuscript
Data collection	Not relevant for this manuscript
Outcomes	Not relevant for this manuscript

Dual use research of concern

Policy information about dual use research of concern

Hazards

Could the accidental, deliberate or reckless misuse of agents or technologies generated in the work, or the application of information presented in the manuscript, pose a threat to:

No	Yes	
☒	☐	Public health
☒	☐	National security
☒	☐	Crops and/or livestock
☒	☐	Ecosystems
☒	☐	Any other significant area

Experiments of concern

Does the work involve any of these experiments of concern:

No	Yes	
☒	☐	Demonstrate how to render a vaccine ineffective
☒	☐	Confer resistance to therapeutically useful antibiotics or antiviral agents
☒	☐	Enhance the virulence of a pathogen or render a nonpathogen virulent
☒	☐	Increase transmissibility of a pathogen
☒	☐	Alter the host range of a pathogen
☒	☐	Enable evasion of diagnostic/detection modalities
☒	☐	Enable the weaponization of a biological agent or toxin
☒	☐	Any other potentially harmful combination of experiments and agents

Plants

Seed stocks	Not relevant for this manuscript
Novel plant genotypes	Not relevant for this manuscript
Authentication	Not relevant for this manuscript

ChIP-seq

Data deposition

☐ Confirm that both raw and final processed data have been deposited in a public database such as GEO.

☐ Confirm that you have deposited or provided access to graph files (e.g. BED files) for the called peaks.

Data access links<br>*May remain private before publication.*	Not relevant for this manuscript
Files in database submission	Not relevant for this manuscript
Genome browser session<br>(e.g. UCSC)	Not relevant for this manuscript

Methodology

Replicates	Not relevant for this manuscript
Sequencing depth	Not relevant for this manuscript
Antibodies	Not relevant for this manuscript
Peak calling parameters	Not relevant for this manuscript
Data quality	Not relevant for this manuscript
Software	Not relevant for this manuscript

Flow Cytometry

Plots

Confirm that:

☐ The axis labels state the marker and fluorochrome used (e.g. CD4-FITC).

☐ The axis scales are clearly visible. Include numbers along axes only for bottom left plot of group (a 'group' is an analysis of identical markers).

☐ All plots are contour plots with outliers or pseudocolor plots.

☐ A numerical value for number of cells or percentage (with statistics) is provided.

Methodology

Sample preparation	Not relevant for this manuscript
Instrument	Not relevant for this manuscript
Software	Not relevant for this manuscript
Cell population abundance	Not relevant for this manuscript
Gating strategy	Not relevant for this manuscript

☐ Tick this box to confirm that a figure exemplifying the gating strategy is provided in the Supplementary Information.

Magnetic resonance imaging

Experimental design

Design type	Not relevant for this manuscript
Design specifications	Not relevant for this manuscript
Behavioral performance measures	Not relevant for this manuscript

Acquisition

Imaging type(s)	Not relevant for this manuscript
Field strength	Not relevant for this manuscript
Sequence & imaging parameters	Not relevant for this manuscript
Area of acquisition	Not relevant for this manuscript

Diffusion MRI ☐ Used ☐ Not used

Preprocessing

Preprocessing software	Not relevant for this manuscript
Normalization	Not relevant for this manuscript
Normalization template	Not relevant for this manuscript
Noise and artifact removal	Not relevant for this manuscript
Volume censoring	Not relevant for this manuscript

Statistical modeling & inference

Model type and settings	Not relevant for this manuscript
Effect(s) tested	Not relevant for this manuscript

Specify type of analysis: ☐ Whole brain ☐ ROI-based ☐ Both

Statistic type for inference (See Eklund et al. 2016)	Not relevant for this manuscript
Correction	Not relevant for this manuscript

Models & analysis

n/a	Involved in the study
☐	☐ Functional and/or effective connectivity
☐	☐ Graph analysis
☐	☐ Multivariate modeling or predictive analysis

Functional and/or effective connectivity	Not relevant for this manuscript
Graph analysis	Not relevant for this manuscript
Multivariate modeling and predictive analysis	Not relevant for this manuscript
